# Vector Selection of a Quadripolar Left Ventricular Pacing Lead Affects Acute Hemodynamic Response to Cardiac Resynchronization Therapy: A Randomized Cross-Over Trial

**DOI:** 10.1371/journal.pone.0067235

**Published:** 2013-06-24

**Authors:** Stefan Asbach, Maximilian Hartmann, Tobias Wengenmayer, Erika Graf, Christoph Bode, Juergen Biermann

**Affiliations:** 1 University Heart Center Freiburg, Germany; 2 Institute of Medical Biometry and Medical Informatics, University Medical Center, Freiburg, Germany; University of Illinois at Chicago, United States of America

## Abstract

**Background:**

A suboptimal left ventricular (LV) pacing site may account for non-responsiveness of patients to cardiac resynchronization therapy (CRT). The vector selection of a novel quadripolar LV pacing lead, which was mainly developed to overcome technical issues with stimulation thresholds and phrenic nerve capture, may affect hemodynamic response, and was therefore assessed in this study. (German Clinical Trials Register DRKS00000573).

**Methods and Results:**

Hemodynamic effects of a total of 145 LVPCs (9.1 per patient) of CRT devices with a quadripolar LV lead (Quartet™, St. Jude Medical) were assessed in 16/20 consecutive patients by invasive measurement of LV+dP/dt_max_ at an invasively optimized AV-interval in random order. Optimal (worst) LVPCs per patient were identified as those with maximal (minimal) %change in LV+dP/dt_max_ (%ΔLV+dP/dt_max_) as compared to a preceding baseline. LV+dP/dt_max_ significantly increased in all 145 LVPCs (p<0.0001 compared to baseline) with significant intraindividual differences between LVPCs (p<0.0001). Overall, CRT acutely augmented %ΔLV+dP/dt_max_ by 31.3% (95% CI 24%–39%) in the optimal, by 21.3% (95% CI: 15%–27%) in the worst and by 28.2% (95% CI: 21%–36%) in a default distal LVPC. This resulted in an absolute additional acute increase in %ΔLV+dP/dt_max_ of 10.0% (95% CI: 7%–13%) of the optimal when compared to the worst (p<0.0001), and of 3.1% (95% CI: 1%–5%) of the optimal when compared to the default distal LVPC (p<0.001). Optimal LVPCs were not programmable with a standard bipolar lead in 44% (7/16) of patients.

**Conclusion:**

The pacing configuration of a quadripolar LV lead determinates acute hemodynamic response. Pacing in the individually optimized configuration gives rise to an additional absolute 10% increase in %ΔLV+dP/dt_max_ when comparing optimal and worst vectors.

## Introduction

Cardiac resynchronization therapy (CRT) has been shown to improve symptoms, quality of life, exercise capacity, and cardiac function and to reduce all-cause and heart failure morbidity and mortality in patients with heart failure, reduced ejection fraction, and cardiac dyssynchrony [1]–[6]. Therefore, in current guidelines [7], CRT is indicated for patients with depressed left ventricular (LV) function, NYHA class II–IV heart failure and a wide QRS complex. However, in the aforementioned large clinical trials, a large proportion of patients did not respond to therapy. This failure to respond may be due to inappropriate patient selection, progressive structural damage to the heart and/or suboptimal placement of the LV lead. Especially the latter has been subject to clinical [8] and experimental [9] studies, trials evaluating a multitude of endocardial pacing sites [10]–[12] and retrospective analyses of large multicenter trials [13], [14]. Inconsistent findings suggest that the optimal pacing site may be specific to each patient and needs individual assessment. In line with this hypothesis, it has recently been shown that individually targeted LV stimulation, as defined by echocardiographic evaluation, can improve results of CRT [15].

While definition of non-response is matter of ongoing debate [16], treatment usually involves echocardiographic evaluation and modification of AV- and VV- intervals.

**Figure 1:**
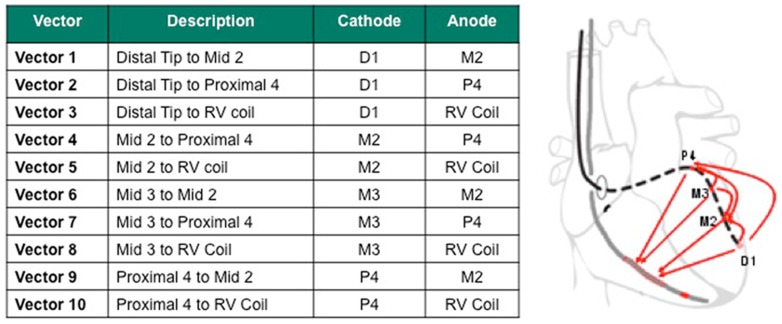
Possible left ventricular pacing configurations - Nomenclature and LVPCs programmable with the quadripolar LV lead.

A novel quadripolar LV lead which offers ten left ventricular pacing configurations (LVPCs) has recently been shown to overcome technical issues with phrenic nerve capture and stimulation thresholds [17]–[19]. With a distance of 4.7 cm between the distal and proximal electrodes, it allows stimulation of the ventricular myocardium along the selected tributary of the coronary sinus (CS) from a distal to a more basal region (Figure 1) and therefore offers additional options to modify device settings for hemodynamic reasons. However, hemodynamic consequences of these different LVPCs are largely unknown. Real life data has already shown that non-traditional LVPCs (i.e. LVPCs not programmable with standard bipolar leads) are used in a large number of patients, and it has been speculated that the choice is not only affected by pacing thresholds, but also by the perceived hemodynamic response [19].

We hypothesized that the hemodynamic response differs according to which LVPC is used and therefore affects response to CRT in the individual. We assessed this hypothesis by evaluating the acute hemodynamic response by invasive measurement of LV +dP/dt_max_ in all possible pacing configurations in patients with clinical indication for CRT and by calculating the %change in mean LV+dP/dt_max_ as compared to the preceding baseline (%ΔLV +dP/dt_max_).

## Methods

Between October 2010 and February 2012, twenty consecutive patients scheduled for implantation of a CRT device at University Medical Center Freiburg met inclusion criteria and were enrolled in the study. Written informed consent was obtained. The study protocol was approved by the local ethics committee. Inclusion criteria included heart failure NYHA II-IV despite optimal medical therapy, LV ejection fraction ≤ 35%, left bundle branch block with QRS width >130 ms and sinus rhythm at the time of implantation. The study is registered at the German Clinical Trials Registry (DRKS00000573).

### Device Implantation

Device implantation was performed in the cardiac catheterization laboratory following a standard procedure. Briefly, after pocket preparation, the subclavian vein was used for vascular access. The ICD shock lead was screwed in the right ventricular (RV) apex in all patients. Subsequently, after an angiogram of the coronary sinus (CS) and its tributaries was performed, a lateral or posterolateral vein was accessed with a guide wire, over which the quadripolar LV lead (Quartet™, St. Jude Medical) was advanced until a stable wedge position was achieved. The atrial lead was screwed into the area of the right atrial appendage. All leads were sutured to the sleeve and attached to the CRT device (Promote Quadra™, St. Jude Medical), which was placed into the prepared pocket. Device interrogation had to confirm stable and adequate lead measurements before the wound was closed. The device was programmed to VVI-40 back-up pacing until optimization.

### Optimization

Invasive optimization was scheduled for the working day following device implantation. In the catheterization laboratory, the Pressure Wire® Certus (St. Jude Medical) was placed in the left ventricle via a 5F femoral sheath and a guiding catheter, and then connected to the electronic control unit (RADI Analyzer® Xpress, St. Jude Medical) and a personal computer for real-time assessment and storage of LV +dP/dt_max_ (PhysioMon© software version 2.02, Radi Medical Systems).

First, the optimal AV delay was assessed by measurement of LV +dP/dt_max_ with the CRT-ICD programmed to a functional VDD modus with AV delays gradually increased from 40 to 300 ms (or appearance of intrinsic conduction) in 20–25 ms steps. These measurements were conducted with the LV lead programmed to a distal (D1M2) and proximal (P4M2) LVPC to exclude interdependence of AV delay and LVPCs. Subsequent biventricular pacing measurements were conducted at the individual optimal AV delay with simultaneous biventricular pacing. No VV-optimization was performed. All measurements started with a 120 s baseline period, followed by 120 s of VDD RV pacing. To minimize confounding effects, all possible LV lead configurations were assessed in random order as outlined below, each active for 120 s and each preceded by a 120 s baseline reference period without pacing to account for potential hemodynamic alterations over time. The optimal and worst pacing sites were defined as the sites which yielded the highest (lowest) value for mean LV +dP/dt_max_ expressed as percent of the mean LV +dP/dt_max_ in the preceding baseline period without pacing (%ΔLV +dP/dt_max_). Optimal versus worst and optimal versus default distal (D1M2) LVPC were compared per patient. The latter will be referred to as “distal” in the text and was chosen because it is the default programming of the device.

### Statistical Analysis

After informed consent, the sequence of biventricular pacing configurations was assigned from a computer-generated randomization list by staff otherwise uninvolved, to guarantee treatment concealment. A Williams design was planned to exclude order effects, so that each pairwise sequence of pacing configurations would occur exactly twice in the 20 patients.

For each patient, absolute LV +dP/dt_max_ measurements in all different LVPCs over the respective 120 s measuring periods were compared to the preceding baseline and to RV only pacing periods using the unpaired t-test. The distributions of %ΔLV +dP/dt_max_ measurements in all LVPCs were compared by intra-individual ANOVA for each patient. Here, a) differences between all interventions were investigated using the F-test for the overall effect of intervention and b) differences between pairs of interventions were investigated via application of the Bonferroni-correction to all possible pairwise comparisons. Test results are reported for optimal versus worst and optimal versus default distal (D1M2) LVPC per patient. Analyses in the entire sample of patients comprised the following. Stability of mean baseline LV +dP/dt_max_ over time was investigated in a mixed model for repeated measures. Mean values for %ΔLV +dP/dt_max_ per LVPC were compared using the paired t-test. Contingency tables were analysed by Fisher’s exact test. All tests were two-sided with significance level α = 5%. Analysis was performed using GraphPad Prism, version 5.0c, ©GraphPad Software, Inc., and the Statistical Analysis System version 9.2, SAS Institute Inc.

## Results

### Implantation and Optimization

In all 20 patients, the device was implanted without acute surgical complications. In all patients, a posterolateral position of the quadripolar LV lead could be achieved. In one patient, an increase of pacing threshold of the RV lead was noted one the day after surgery and was subsequently repositioned thereafter.

**Figure 2:**
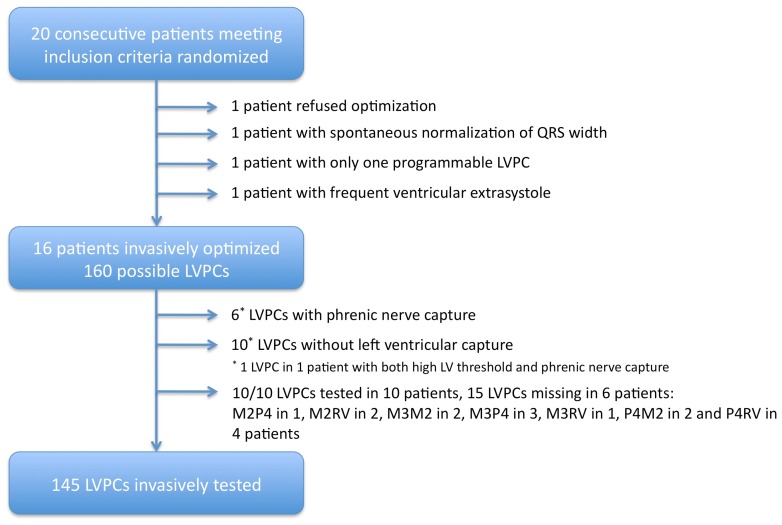
Study flow chart – Distribution of the tested LVPCs.

After informed consent, one patient refused invasive testing, one had spontaneous recovery of left bundle branch block to a narrow QRS complex, and one patient had only one programmable LVPC and was therefore not invasively optimized. An additional patient had to be excluded because of frequent ventricular extrasystole precluding invasive testing. The remaining 16 patients constituted the study group and were invasively optimized on the working day following CRT device implantation (Figure 2). Please refer to Table 1 for patient characteristics. Out of the theoretically possible 160 LVPCs (10 per patient), 15 could not be performed: in 10 cases because of exit block and in 6 cases because of phrenic nerve capture (one stimulation site had both, high threshold and phrenic nerve capture). Thus, LV +dP/dt_max_ was assessed in 145 LVPCs (Figure 2). The mean optimal AV-delay was 91.3±26.3 ms and did not differ intraindividually when assessed with the LV lead programmed to the D1M2 or the P4M2 LVPC.

**Table 1:** Patient characteristics.

Patient characteristics (n = 16)	
Age (years)	67.7±9.4
Sex (male/female)	4/12
Ischemic/dilated cardiomyopathy	4/12
NYHA functional class (II/III/IV)	7/9/0
LBBB (%)	100
QRS width (ms)	167.9±19.8
LV EF (%)	23.8±4.3
LVEDD (mm)	66.1±8.3
LVESD (mm)	52.6±8.5
proBNP (pg/ml)	4009.5±5016.1

LBBB: left bundle branch block, LV EF: left-ventricular ejection fraction, LVEDD: left ventricular end-diastolic diameter, LVESD: left ventricular end-systolic diameter.

### Intraindividual Effects of LVPCs

In each patient, all LVPCs improved LV +dP/dt_max_ significantly as compared to the preceding baseline without stimulation (p<0.0001 for each of the 145 comparisons). Likewise, most LVPCs improved LV +dP/dt_max_ significantly as compared to RV (VDD, AV-optimized) pacing (p<0.05 for 140/145 comparisons and p = ns for 5/145 comparisons).

We found no evidence of carry-over: Changes of mean baseline LV +dP/dt_max_ over time were neither statistically significant (p = 0.107) nor of a relevant magnitude (3% change from minimum 846.8±111.8 mmHg/s to maximum 871.8±112.1 mmHg/s). Optimal and worst LVPCs were identified as outlined above. Here, biventricular pacing acutely augmented LV +dP/dt_max_ from 838.6±108.5 to 1097.2±161.5 mmHg/s in the optimal (p<0.0001), from 860.2±109.4 to 1040.3±143.9 mmHg/s in the worst (p<0.0001) and from 857.5±117.4 to 1085.1±160.4 mmHg/s in the distal LVPC (p<0.0001).

Overall effects between LVPCs were significantly different (p<0.0001 in every patient).

**Figure 3:**
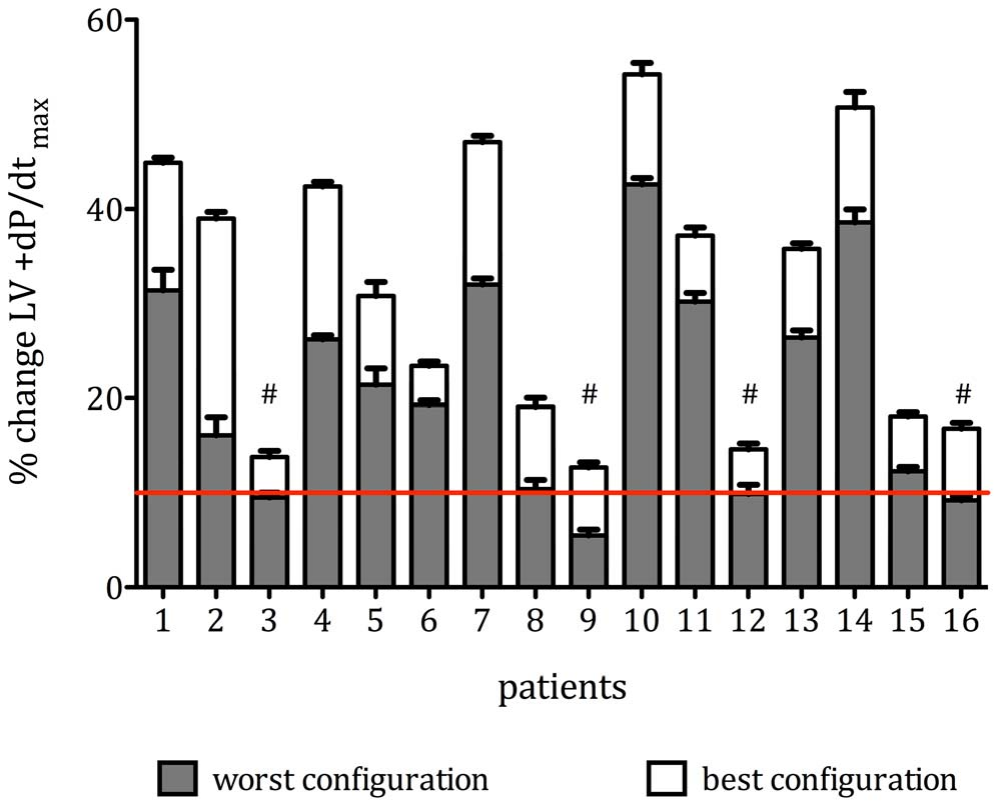
Comparison of optimal and worst LVPCs - Individual results in increment in mean %ΔLV +dP/dt_max_ (±95% confidence interval) in all patients in the optimal (white) and worst (grey) pacing configuration (p<0.0001 for all intraindividual differences). The red line indicates a 10% increase in LV +dP/dt_max_, which has been proposed as a cut-off value to separate responders from non-responders (20). By this definition, individually tailoring the optimal pacing configurations in 4 patients (marked #) transformed non-responders into responders.

**Figure 4:**
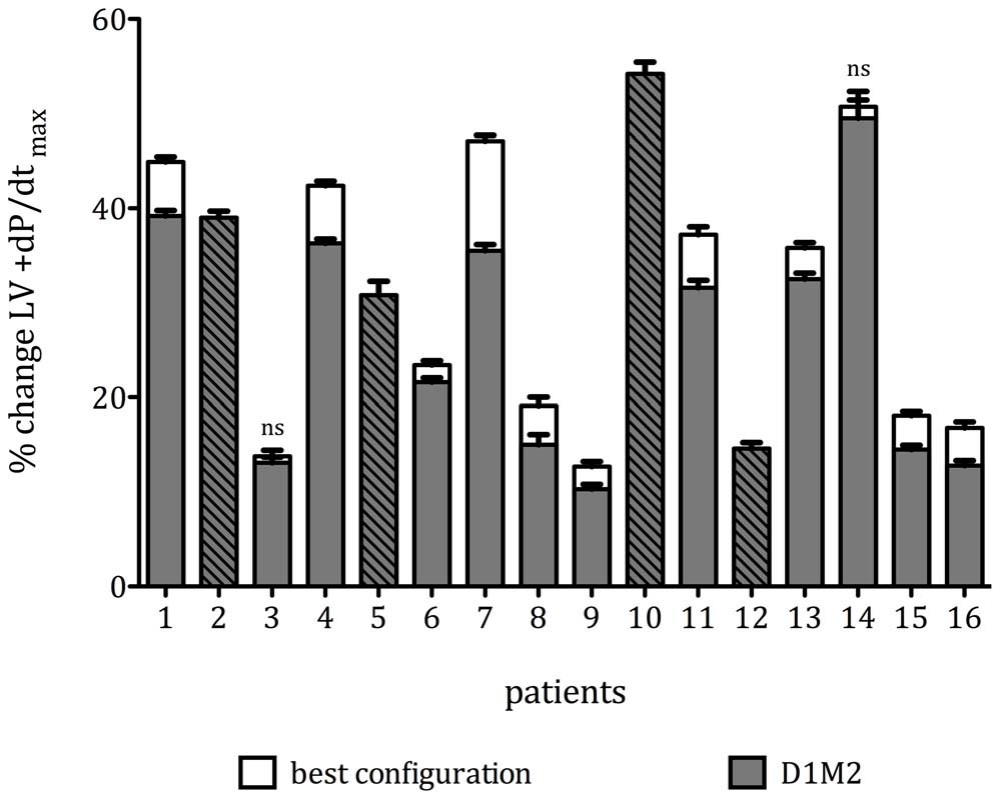
Comparison of optimal and distal LVPCs - Individual results in increment in mean %ΔLV +dP/dt_max_ (±95% confidence interval) in all patients in the optimal (white) and D1M2 (grey) pacing configuration (p<0.0001 for all intraindividual differences except for patients 3 and 14 (ns). Striped bars indicate those 4 patients in whom D1M2 exhibited maximal increase in %ΔLV +dP/dt_max_.

After application of correction for multiple comparisons, we found (by intraindividual comparison of all LVPCs as %ΔLV +dP/dt_max_) significant differences between optimal and worst LVPCs in all 16 patients (p<0.0001, Figure 3) and significant differences between optimal and distal LVPCs in 10/12 patients (p<0.0001, Figure 4, 4 patients had D1M2 as optimal LVPC). A difference in %ΔLV +dP/dt_max_ of more that 10% was found in 6/16 (37.5%) of patients.

**Figure 5:**
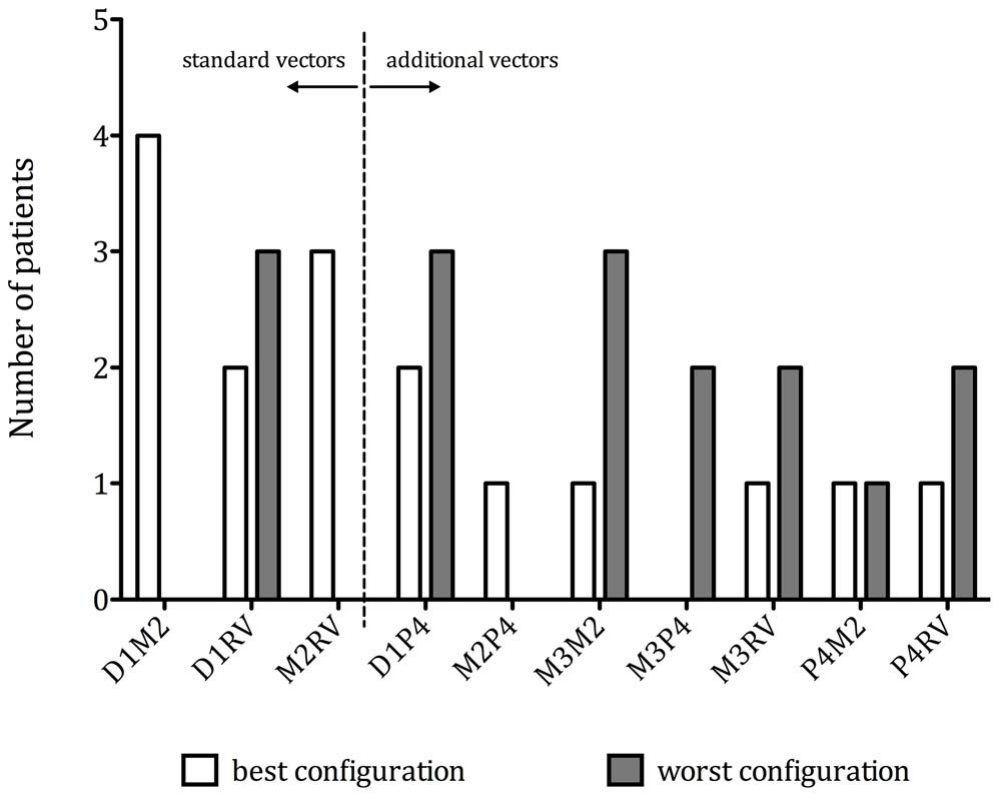
Distribution of optimal (white) and worst (grey) pacing configurations among study patients - Left three columns show configurations programmable with a standard bipolar lead.

Distal LVPCs (D1 and M2 as cathode) more often showed optimal increment in %ΔLV +dP/dt_max_ than proximal LVPCs (M3 and P4 as cathode) (12 (75%) versus 4 (25%), p = 0.012), whereas proximal LVPCs by trend more often showed worst increment in %ΔLV +dP/dt_max_ (10 (63%) versus 6 (37%), p = 0.289). Please refer to Figure 5 for distribution of optimal and worst LVPCs.

When classifying by standard (D1M2, D1RV, M2RV) or non-standard (remaining 7) LVPCs, in 9 patients (56%) standard vectors and in 7 patients (44%) non-standard vectors resulted in optimal %ΔLV +dP/dt_max_ (Figure 5, p = 0.724). Two out of 4 patients with ischemic (50%) and 2 out of 12 patients with non-ischemic (16.7%) cardiomyopathy derived most benefit from a proximal (M3 or P4 as cathode) stimulations site (p = 0.245).

When using a prespecified threshold of 10% increase in %ΔLV +dP/dt_max_ as previously proposed [20], in 4 patients (25%) individually tailoring the optimal pacing configuration transferred non-responders into responders and thereby increased the overall response rate from 75% to 100% (Figure 3). Of note, in 3 of these 4 patients, a non-standard and in 2 of these 4 patients, a more proximal pacing configuration yielded optimal results.

### Interindividual Effects of LVPCs

**Figure 6:**
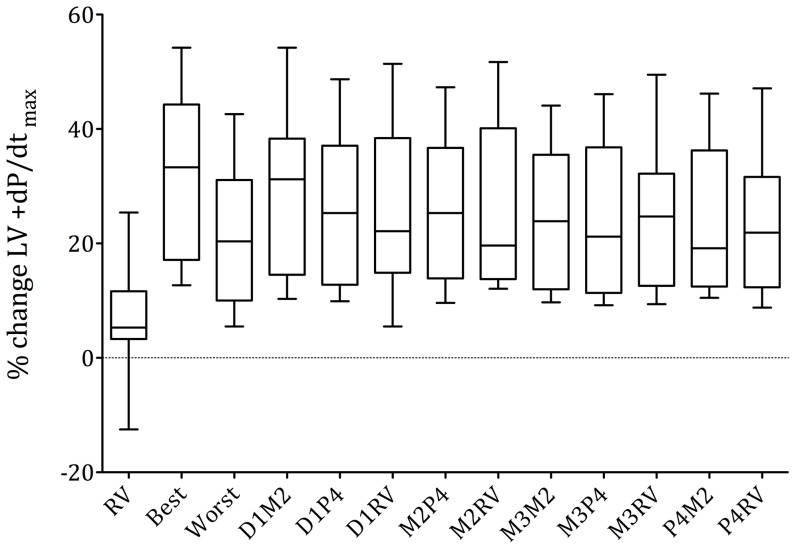
Averaged results over all patients - Box plots of %ΔLV +dP/dt_max_, of all LVPCs, averaged over all 16 patients (5–95% percentile). RV stimulation consistently produced the least increase in LV +dP/dt_max_. No differences were found between LVPCs with the same cathode.

Overall, biventricular pacing acutely augmented %ΔLV +dP/dt_max_ by 31.3% (95% CI: 24%–39%) in the optimal, by 21.3% (95% CI: 15%–27%) in the worst and by 28.2% (95% CI: 21%–36%) in the distal LVPC. This resulted in a mean 10.0±5.1% (95% CI: 7%–13%) additional acute increase in %ΔLV +dP/dt_max_ of the optimal when compared to the worst (p<0.0001) and a 3.1±3.1% (95%CI: 2%–5%) additional acute increase in %ΔLV +dP/dt_max_ of the optimal when compared to the distal LVPC (p<0.001). All LVPCs were inferior to the mean of the best LVPC (p<0.05) and superior to the mean of the worst LVPC (p<0.01). There were no significant differences between LVPCs with the same cathode. Figure 6 illustrates the acute response to biventricular pacing in all possible LVPCs averaged over all patients.

### Effects of Right-ventricular Pacing

Intraindividually, RV VDD, AV-optimized pacing produced a significant increase in LV +dP/dt_max_ in 15/16 patients (p<0.005 for each comparison). Here, RV pacing acutely augmented LV +dP/dt_max_ from 873.7±115.7 to 942.4±124.9 mmHg/s. In one patient, a significant decrease in LV +dP/dt_max_ was noted (p<0.0001). Overall, this resulted in an increase of 6.7±8.1% as compared to baseline.

## Discussion

This study presents the first systematic, randomized investigation of acute hemodynamic effects of all different LVPCs of a commercially available quadripolar LV pacing lead. Our main findings are that (1) acute hemodynamic effects are determined by LVPCs leaving space for an additional average 10% absolute increase in %ΔLV +dP/dt_max_ when comparing optimal and worst LVPCs with (2) interindividual variations in response with respect to the optimal LVPC, which (3) in 44% of patients were LVPCs not programmable with a standard bipolar lead.

These findings may have implications on how to deliver optimal cardiac resynchronization therapy with a growing number of opportunities in modern CRT devices, may be of special value in the treatment of non-responders, and may form a concept on future LV lead developments.

### Impact of LV Pacing Site

The initial evaluation of LV pacing sites by Butter et al. [8] revealed – by comparing two pacing sites - that LV lateral free wall pacing was superior to anterior pacing and formed the currently followed concept of lateral or posterolateral LV pacing for most patients undergoing CRT therapy. This concept, however, has recently been challenged by animal studies in failing heart showing that lateral, but more anterior and apical pacing sites produced best CRT effect [9], studies in humans involving evaluation of multiple endocardial pacing sites in patients with ischemic [10], [12] and non-ischemic cardiomyopathy [11], [12], as well as retrospective analyses of large multicenter trials [13], [14]. In accordance with these results, we found significant interindividual differences with respect to the optimal choice of the LVPC: We found differences of >10% in absolute %ΔLV +dP/dt_max_ in 38% of our patients with differences up to 22.9% in the individual.

In line with previous published data [10]–[12], the optimal configuration seems to be specific to each individual with the default distal LVPC being the optimal only in 4/16 patients. Therefore, it is easily conceivable that studies that evaluated CRT response at different sites within single CS tributaries did not find systematic, but substantial intraindividual differences with respect to the optimal pacing site [21], [22].

Nowadays, epicardial LV stimulation via the CS is standard of care, even though, in the individual patient, sites not accessible via the CS may yield better response. This was specifically demonstrated by accessing the LV endocardium in 35 patients with non-ischemic cardiomyopathy [11] and 11 patients with ischemic cardiomyopathy [10]. Derval et al. [11] found that (LV-only) pacing from within the CS augmented LV +dP/dt_max_ by 15±23% to a significantly lower extent than the best endocardial site (31±26%). Spragg et al. [10] found an increase in LV +dP/dt_max_ by CS pacing of 13% versus an average 36% increase with endocardial LV pacing in 7 patients with ischemic cardiomyopathy. The optimal pacing site here was often found in the extreme base of the LV. This site in most cases could not be stimulated with proximal vectors in our study, since, for stability reasons, the LV electrode was advanced until a distal wedge position was achieved. Possibly, further developments of LV multipolar electrodes might overcome this issue by adding some or increasing the distance between electrodes. However, as we now could show that individually programming a quadripolar lead adds an additional absolute 10% of %ΔLV +dP/dt_max_ increase, this might already help to overcome some of the restraints linked to pacing in distal CS tributaries. It must also be acknowledged, that it is a complex and time-consuming procedure to individually assess a multitude of pacing sites from the endocardium, there is limited experience with permanently pacing the LV from the endocardial side [23], [24], and optimal pacing sites may vary over time with changing activation patterns and/or cardiac geometry. The latter might at least in part be overcome by simple reprogramming of a multipolar electrode rather than by revision of an implanted endocardial lead.

### Strategies to Define Optimal LV Pacing Site

As outlined above, results from many trials performed have shown that the location of the optimal pacing site shows significant interindividual variability and with a resulting need for individual assessment. Acute response to CRT as measured by LV +dP/dt_max_ has been shown to be predictive of long-term response [20], however, this issue is still matter of debate [25], and, in fact, there is also evidence against this assumption [26]. Echocardiographically tailored stimulation of the LV seems to be able to increase the effect of CRT [15]. The TARGET Study [15] revealed that, as defined by echocardiography, about one third of optimal stimulation sites are located basally. In line with this, in our population 25% of patients exhibited best response when programmed to a more basally located LVPC, a proportion that might even increase if only patients with ischemic cardiomyopathy are regarded [10]. Stable lead position at these sites is hard to reach with standard, non-active fixation leads and may thus be more efficiently treated with proximal electrodes of a multipolar lead. A multipolar lead may also be reprogrammed with respect to stimulation site if the optimal pacing spot changes over time [27], [28].

### Study Limitations

The study is limited by the small number and the heterogeneity of the included patients, therefore, measures, such as randomization of pacing sequence and multiple and lengthy baseline periods have been taken to control confounding effects. This extensive protocol might not be suitable for routine implementation in clinical practice. It must also be acknowledged that additional confounding effects, such as the severity of mitral regurgitation [29] and the amount of myocardial scar [30] affect CRT response and where not systematically assessed in this study. As noted above, acute hemodynamic response may not translate into chronic benefitial effects and may not be measurable with clinical endpoints.

### Conclusions

The LVPC of a quadripolar LV lead determinates acute hemodynamic response. Pacing in the individually optimized LVPC gives rise to an average absolute 10% increase in %ΔLV +dP/dt_max_ when comparing optimal and worst vectors. Future studies need to be performed to evaluate the short- and long-term outcome of individualised pacing along the quadripolar lead.
